# Diagnosis and Treatment of Benign Bleeding Disorders

**DOI:** 10.6004/jadpro.2016.7.3.17

**Published:** 2016-04-01

**Authors:** Ravi Krishnadasan

**Affiliations:** University of Arizona, Tucson, Arizona

Many common bleeding disorders will be revealed through a careful examination of patient history and a mixing study, according to Ravi Krishnadasan, MD, of the University of Arizona, Tucson, Arizona.

Benign bleeding disorders are understood in the context of the two main components of hemostasis: primary and secondary hemostasis. Primary hemostasis is the formation of the platelet plug, which involves platelets, von Willebrand factor, and binding to collagen. Secondary hemostasis involves factors such as factors IX, X, XI, and XII that form the protective fibrin clot as a result of a "coagulation cascade." Platelets are involved in both types of hemostasis.

At JADPRO Live at APSHO, Dr. Krishnadasan described common bleeding disorders and offered clinical pearls on evaluation and treatment. "The first and most important step is taking a careful patient history," he stressed. "Decipher whether the pattern of symptoms is consistent with primary or secondary hemostasis."

Clinicians should ask about previous symptoms, history of procedures and transfusions, occurrence of trauma vs. spontaneous bleeds, time and age of onset, and family history. For women, estrogen exposure and pregnancy are important factors; some bleeding disorders, such as menorrhagia, improve in the presence of high estrogen.

Microhemorrhage (including mucocutaneous bleeding [gums, nose]), bruising, and petechiae, are usually related to primary hemostasis. Such patients often present after experiencing heavy bleeding after a tooth extraction, for example.

On the other hand, macrohemorrhage—large bleeds into the joints and muscles or widespread bruising—is associated with secondary hemostasis. One such condition is hemophilia (factor VIII or IX deficiency).

## LABORATORY TESTING

Clinicians should know which laboratory tests will be informative. In primary hemostasis, platelet function testing is important, but a bleeding time assay is no longer routine. Rather, platelet aggregation is the recommended test for platelet function. Platelet function assay (PFA-100) and thromboelastrograms are also increasingly used in some situations.

"It is important that when you do a test for platelet function, the patient should be off NSAIDS (nonsteroidal anti-inflammatory drugs) and aspirin for at least 7 days, as they affect platelet function," Dr. Krishnadasan emphasized.

von Willebrand disease (VWD) is the most common bleeding disorder, affecting an estimated 1% of the US population. Three initial screening tests are used: von Willebrand antigen, von Willebrand activity, and factor VIII activity. They are followed by more specific second-tier tests when a deficiency is suspected. The standard tests for secondary hemostasis include prothrombin time (PT), activated partial thromboplastin time (aPTT), mixing study, factor activities, factor XIII, factor inhibitor, and lupus anticoagulant studies (which are associated with clotting, not bleeding).

"The mixing study is the first and most important test," he said. Mixing studies are performed on plasma to distinguish factor deficiencies from factor inhibitors, such as lupus anticoagulant, or specific factor inhibitors, such as antibodies directed against factor VIII. Mixing studies take advantage of the fact that factor levels that are 50% of normal should give a normal PT or PTT. The clinician mixes a sample of the patient’s serum with normal serum and observes whether the level corrects immediately or partially corrects.

## TYPICAL CASE IN THE CLINIC

Dr. Krishnadasan described the type of patient he frequently encounters: a young woman presenting for surgery whose aPTT was elevated on preoperative lab tests. "You see the patient Monday, and her surgery is Friday. This is common in our office."

"My first step is to get a bleeding history," he said. He generally runs through a list of questions (see the Table) to get a background on the bleeding or clotting issues. His next step is to get a mixing study, which will help him determine whether the problem is a factor deficiency or inhibitor. If no concerning issues emerge, and the patient has no history of bleeding or clotting, Dr. Krishnadasan would recommend that she undergo the surgery under close observation and with appropriate prophylaxis for deep-vein thrombosis.

**Table 1:**
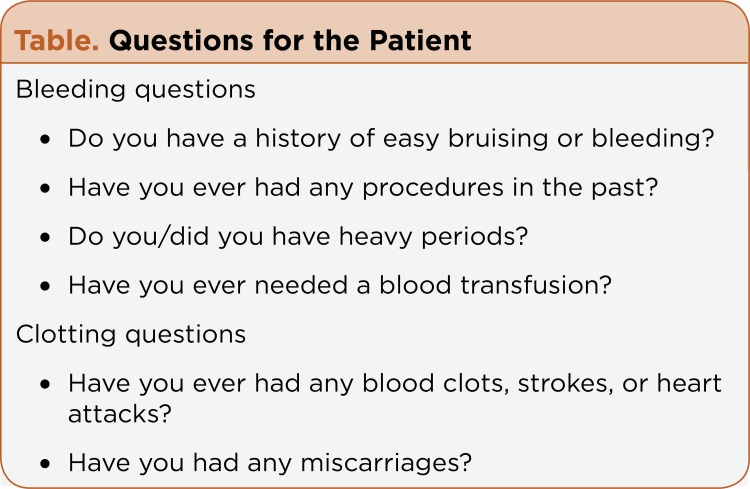
Questions for the Patient

## von WILLEBRAND DISEASE AND PLATELET DISORDERS

von Willebrand disease (VWD) is a genetic disorder caused by a missing or defective von Willebrand factor, a clotting protein. There are three types of inherited VWD. Types 1 (up to 75% of cases) and 3 are quantitative deficiencies, whereas type 2 reflects a qualitative deficiency in antigen level. A fourth type that is not inherited is acquired VWD.

Mild type 1 disease is treated with desmopressin acetate (DDAVP), which can "bump up" von Willebrand factor levels before medical procedures; short-acting antifibrinolytic drugs (aminocaproic acid and tranexamic acid) can also do this. For type 3 disease, plasma-derived products are used. Oral contraceptives can be given as well. 

Essential thrombocytosis is generally associated with clotting (thromboses), but in some cases it is associated with bleeding. Persons with extremely high platelet counts can have an acquired von Willebrand defect; aspirin should be used with caution.

Platelets < 100,000/L may indicate immune thrombocytopenia (ITP). Most primary cases are idiopathic; secondary ITP can be caused by drugs and viral infection.

"There is no good test for ITP. It is a diagnosis of exclusion," indicated Dr. Krishnadasan. Testing for antiplatelet antibodies in the workup of ITP "is a waste of money," he maintained. "The results won’t change your management, so don’t use this test."

There are a number of evidence-based approaches to treating ITP. The standard of care is a steroid alone (prednisone or dexamethasone) for patients who are not actively bleeding. He prefers prednisone (20 mg) and noted that dexamethasone requires multiple treatments. Splenectomy cures two-thirds of patients but is best reserved until patients have had a chance to recover. Eltrombopag (Promacta) and romiplostim (Nplate) are also part of the armamentarium. Intravenous immunoglobulin (IVIG) is useful prior to surgery and, unlike prednisone, is not associated with wound-healing problems. Dr. Krishnadasan advised reserving rituximab (Rituxan) for second-line use.

"There are a number of treatments for ITP, and there is no right one," he commented.

## HEMOPHILIA

Hemophilia—factor VIII or factor IX deficiency—ranges from mild to severe, depending on the level of factor present. "You only need 1% to 2% of factor. At this level, you will have bleeding only after trauma," Dr. Krishnadasan said. Because bleeding episodes are rare in mild cases, such patients may not be diagnosed until later in life.

The treatment is factor replacement. In the presence of antibodies against factor VIII, bypassing agents such as activated factor VIIa or FEIBA (anti-inhibitor coagulant complex; activated prothrombin complex concentrate) must be used. Treatment may be "on demand" or prophylactic.

Additional factor deficiencies include factor XI and factor XII deficiency (in which case aPTT is elevated), factor VII deficiency, and vitamin K deficiency (in which case PT is elevated). These factor deficiencies can be revealed in mixing studies.

Dr. Krishnadasan emphasized the importance of recognizing acquired hemophilia, which often reveals itself by partial correction with prolongation of aPTT on mixing studies and is usually a factor VIII deficiency. "Don’t miss these patients," he urged. "Rates of morbidity and mortality are high, even with the best treatment."

These patients may often include elderly patients who present with extensive bruising or bleeding, pregnant females, patients with autoimmune or lymphoproliferative disease, and patients receiving interferon for hepatitis C. 

"First stop the bleeding, and check inhibitor titers. If they are low (< 5 Bethesda units [BUs]), give high doses of factor VIII or IX. If they are high (> 5 BUs), give a bypassing agent," Dr. Krishnadasan advised. "You are also going to need to suppress antibody production, and for this I use prednisone with cyclophosphamide."

